# The value of brain MRI functional connectivity data in a machine learning classifier for distinguishing migraine from persistent post-traumatic headache

**DOI:** 10.3389/fpain.2022.1012831

**Published:** 2023-01-09

**Authors:** Gina Dumkrieger, Catherine D Chong, Katherine Ross, Visar Berisha, Todd J Schwedt

**Affiliations:** ^1^ Department of Neurology, Mayo Clinic Arizona, Phoenix, AZ, United States; ^2^ Phoenix VA health care system, Veterans Health Administration, Phoenix, AZ, United States; ^3^ Department of Speech and Hearing Science and School of Electrical Computer and Energy Engineering, Arizona State University, Tempe, AZ, United States

**Keywords:** post-traumatic headache (PTH), migraine, fMRI, classification, machine learning

## Abstract

**Background:**

Post-traumatic headache (PTH) and migraine often have similar phenotypes. The objective of this exploratory study was to develop classification models to differentiate persistent PTH (PPTH) from migraine using clinical data and magnetic resonance imaging (MRI) measures of brain structure and functional connectivity (fc).

**Methods:**

Thirty-four individuals with migraine and 48 individuals with PPTH attributed to mild TBI were included. All individuals completed questionnaires assessing headache characteristics, mood, sensory hypersensitivities, and cognitive function and underwent brain structural and functional imaging during the same study visit. Clinical features, structural and functional resting-state measures were included as potential variables. Classifiers using ridge logistic regression of principal components were fit on the data. Average accuracy was calculated using leave-one-out cross-validation. Models were fit with and without fc data. The importance of specific variables to the classifier were examined.

**Results:**

With internal variable selection and principal components creation the average accuracy was 72% with fc data and 63.4% without fc data. This classifier with fc data identified individuals with PPTH and individuals with migraine with equal accuracy.

**Conclusion:**

Multivariate models based on clinical characteristics, fc, and brain structural data accurately classify and differentiate PPTH vs. migraine suggesting differences in the neuromechanism and clinical features underlying both headache disorders.

## Background

Migraine and post-traumatic headache (PTH) often share similar phenotype and most individuals with persistent PTH (PPTH) have a migraine-like phenotype (1, 2). New headaches starting in close temporal relation to traumatic brain injury (TBI) are easy to identify as PTH when pre-existing headaches are not present. However, the differentiation is more difficult when the headaches are longstanding and recollection of the timing of TBI and specific headache symptoms may be unclear. In these situations, when the diagnosis lacks certainty on clinical grounds alone, an objective method to help differentiate PPTH from migraine could be of significant value and increase confidence in the diagnosis.

Recent studies have found disease-specific differences in clinical characteristics and unique differences in brain structure and function underlying both headache disorders (3–6). In a previously published study, we developed a logistic regression classifier based on principal components (PCs) using clinical variables and brain structural data to understand the extent to which PPTH and migraine have disparate pathophysiology. In this study, machine learning classifiers incorporating functional imaging data as well as clinical and structural imaging data were constructed. In addition to clinical utility of differentiating those with migraine from those with PPTH, the classifiers provide an opportunity to learn more about the diseases and what separates them, by discovering patterns not apparent with a simpler analysis such as univariate analysis.

The goal of this current study was to assess whether adding resting state static and dynamic fc measures in these models could improve classification accuracy for differentiating those with migraine from those with PPTH.

## Methods

### Participant eligibility criteria

Male and female study participants ages 18–65 years were recruited from the Phoenix Veterans' Administration (VA) Health Care System and Mayo Clinic Arizona, both sites from which IRB approvals were obtained. Prior to participation, study participants completed written informed consent at the recruitment site. Diagnosis of migraine or PPTH attributed to mild TBI was made according to ICHD-3 beta (8) criteria and assigned by a headache specialist. Migraine subjects were excluded if they had any history of TBI. Individuals with PTH were excluded if they had a history of migraine or a history of moderate or severe TBI.

Thirty-four individuals with migraine and 48 participants with PPTH are included in this study. Subjects from this study have been included in prior publications which have shown differences between migraine and PPTH groups in brain structure (9, 10), fibertract profiles (11), static and dynamic functional connectivity (12), autonomic symptoms (3) and insomnia (4). Extending a classifier based on clinical data and structural imaging data (7), here for the first time we create a migraine vs. PPTH classifier including clinical data, structural and functional imaging data.

In the PPTH group 10 subjects reported one lifetime concussion, 19 reported two, 15 reported 3–10 and four PPTH subjects reported 10 + concussions. Of the group with 10 + concussions two were due to repeated impacts from sports and two were due to military training or active-duty blast-related injuries. In total 10 subjects' most recent concussions were sport related, seven were due to motor vehicle accident, 10 were due to falls and 21 were blast related.

Thirty migraine subjects met the headache frequency criteria for chronic migraine per ICHD-3 beta with the remaining four classified as episodic.

### Clinical data collection

Study participants completed a battery of psychological and cognitive evaluations including the Rey Auditory Verbal Learning Test (RAVLT); immediate and delayed memory recall (13); Trail Making Test (TMT) (14); Beck Depression Inventory (BDI) (15); and State-Trait Anxiety Inventory (STAI); Form Y-1 and Form Y-2 (16). Other symptoms were assessed with Hyperacusis Questionnaire (17); Photophobia Assessment Questionnaire (PAQ) (18); Allodynia Symptom Checklist, (ASC)-12 (19); COMPASS 31 (20); Migraine Disability Assessment Scale (MIDAS) (21); a validated post-traumatic stress disorder checklist (DSM-5) (22); the Pain Catastrophizing Scale (PCS) (23, 24) and a detailed headache questionnaire developed by headache specialists at Mayo Clinic. Patients completed the Ohio State University TBI identification method questionnaire (25). A case report form containing Common Data Elements developed by the National Institute of Neurological Disorders and Stroke (26) and a 22-item Symptom Evaluation Checklist from the Sport Concussion Assessment Tool (SCAT) 5th edition (27) were used to characterize headache and TBI characteristics.

### Brain imaging data

Following questionnaire completion subjects had structural and resting-state functional imaging of the brain. Images were collected on a single 3-Tesla Siemens MAGNETOM (Erlangen, Germany) scanner. 3D T1-weighted sagittal MPRAGE, axial T2-weighted imaging, Diffusion Tensor Imaging (DTI) (non-linear directions and one image without diffusion weighting) and ten minutes of blood oxygenation level dependent (BOLD) resting state imagine data were collected. For the resting state scans, participants were instructed to keep their eyes closed but to remain awake, to relax, and to try to clear their minds.

See appendix for imaging sequence details.

T1 and T2 weighted scans were reviewed by a board certified neuroradiologist. Subjects with presence of gross anatomical abnormalities on imaging (including T2 hyperintensities) were excluded from the final analysis. T1-weighted images were segmented using FreeSurfer version 6.0 (28) using the Desikan-Killiany Atlas.

DTI preprocessing was done using the automated tractography toolbox TRACULA (TRActs Constrained by Underlying Anatomy) (29). Preprocessing steps included image correction, brain extraction, within-subject registration to the individual's T1-weighted image, and co-registration to a template. The distribution of fibertracts was estimated using a Markov Chain Monte Carlo algorithm. A total of 18 fibertracts were extracted.

Resting state data were slice-time and motion corrected, and realigned. Skull and non-brain tissue were removed, data were smoothed, aligned to each subject's own T1-weighted scan, and transformed to the standardized Montreal Neurological Institute (MNI) template. Data were bandpass filtered and signals of no interest and head motion were regressed from the data.

Functional connectivity was assessed using a region of interest approach with 69 regions drawn according to findings from prior literature. Static fc was assessed by finding the correlation between the time series of two regions. Dynamic fc for each region pair was calculated by sliding window correlations with window length of 60 s and overlap of 1 frame. Window-length of 60 s was chosen to be consistent with our previous work (12) and with findings that window-lengths of approximately this length are optimal in the absence of information about the true correlation timescale (30). Regions of interest, detailed imaging parameters and preprocessing details can be found in the appendix material [Supplementary Table S1, Appendix].

### Classification model

Some collected clinical data were not included as variables in the model because the data were directly tied to the classification in a way that would unfairly bias the model and artificially increase the model accuracy or because their inclusion would negatively affect the applicability of the model to other populations. For example, variables related to history of TBI were excluded because every PPTH participant had a previous TBI but history of TBI was an exclusion criterion for the Migraine group. PTSD (which would likely be much higher in the Phoenix VA PPTH population), headache frequency, years with headache, family history of migraine, and presence of aura were also excluded.

In our prior publication, clinical data, structural and fiber tract measures were standardized, converted to principal components and a logistic ridge regression model was fit on the principal components (PC) (7). Leave one out cross-validation was used to assess model performance and the ridge L2 regularization parameter was set within the cross-validation loop. Within each cross validation loop an additional, inner, leave one out cross validation (81-fold) was performed for each candidate regularization parameter. The candidate ridge parameter with the best performance over the inner cross validation loops was chosen as the ridge parameter for the primary, outer, cross validation loop.

In this analysis, the addition of the many (4692) fc derived variables necessitated variable selection prior to model fitting. Welch's *t*-tests were used to identify static and dynamic connectivity variables that showed group differences between migraine and PPTH. The fc variables with significant differences between groups were included as candidate variables for the classification model. Candidate variables were standardized to mean 0, and unit variance. PCs of the standardized data set were found using the PCA function from Python's scikit-learn package. Candidate variables from all data sources were utilized together to create the PCs. These PCs were the feature set for the classification model.

Leave one-out cross-validation was used to estimate model accuracy. Within the cross-validation loop, the preferred L2 penalty (regularization parameter) was determined from a list of candidates. The overall classification model was a logistic regression model with L2 penalty with PCs as the model variables. Candidate ridge parameter values and the use of 65 PCs were chosen for consistency with the previous publication.

### Bias correction

When variable selection is performed outside of a cross-validation loop all data are used to select variables, including the held-out sample. This may result in a biased estimate of model accuracy. To address this, a second approach was taken in which the variable selection was performed inside the cross-validation loop, meaning that the held-out sample was not used in variable selection. This is referred to as the internal model. When the variable selection of fc data was performed within the cross-validation loop, the number of variables used to create the PCs varied by loop, but the number of PCs remained at 65. Within each cross-validation loop the entire set of candidate variables selected within that loop were utilized to create the PCs for that loop.

### Variable importance

Following the model fitting, variable importance in the internal variable selection and PC creation model with fc data was estimated with the following custom metric: first the contribution of each variable to each PC was found. Then the contribution of each PC to the logistic regression was found from the standardized regression coefficients. Each value is a percentage. By multiplying the two it is possible to estimate the contribution of each variable to the model. The sum of the contribution of all variables for one model is 1. Two things must be noted about this metric. First, ridge regression does not permit a coefficient value to be set to 0, meaning that the minimum contribution of each PC to the model is greater than zero. Secondly, the contribution of an individual variable is tied to the contribution of the PCs onto which it projects. Only variables that are excluded during variable selection or which mapped entirely to PCs not passed to the regression model will have a contribution of zero. Mean variable importance over all cross-validation loops was used to rank model importance.

## Results

Information on subject demographics can be found in Table 1. Thirty-four individuals with migraine and 48 PPTH participants were included. There were no significant differences in age between the migraine and PPTH groups (mean(sd): Migraine: 41.7 (10.9); PPTH: 38.1 (10.7); *p* = 0.120). The PPTH group had a higher proportion of males (Migraine: 35.3%; PPTH: 64.6%; *p* = 0.017), lower headache frequency (Migraine: 20.4 (6.2); PPTH: 16.1 (8.6); *p* = 0.015) and less years lived with headache (Migraine: 24.9 (14.4); PPTH: 10.6 (8.0); *p* < 0.001). The PPTH group also had higher scores on the BDI (Migraine: 9.0 (6.0); PPTH: 17.1 (8.9); *p* < 0.001), COMPASS 31 (Migraine: 27.3 (12.9); PPTH: 36.6 (14.4); *p* = 0.006), hyperacusis (Migraine:14.4 (8.5); PPTH:23.3 (10.7); *p* < 0.001) and insomnia (Migraine:18.3 (6.8); PPTH 23.5 (6.6); *p* = 0.002) questionnaires. There were no significant differences between the migraine and PPTH groups in percentage reporting “White/Caucasian” race (Migraine: 94.1%; PPTH: 93.8%; *p* = 1), Hispanic ethnicity (Migraine: 4.2%; PPTH 16.7%; *p* = 0.073), or in ASC-12 (Migraine: 5 (4.1); PPTH: 5.6 (5.2); *p* = 0.895), MIDAS (Migraine: 48.5 (33.5); PPTH: 67.9 (59.8); *p* = 0.305), PCS (Migraine: 19.9 (10.9); PPTH: 24.4 (13.2); *p* = 0.155), state anxiety (Migraine:34.8 (8.9), PPTH: 37.7 (13.4); *p* = 0.498) or trait anxiety (Migraine: 40 (10.3); PPTH: 45 (13.4); *p* = 0.062) scores. Information on income, educational level and other socioeconomic status indicators was not collected.

**Table 1:** Subject Descriptors.

		Migraine	PPTH	*p*-value
		*n*/total, (%)	*n*/total, (%)	
**Sex (male)**		12/34, (35.3%)	31/48, (64.6%)	**0**.**017**
**Aura**		18/34, (52.9%)	23/48, (47.9%)	0.823
**Race**	White/Caucasian	32/34, (94.1%)	45/48, (93.8%)	1
	American Indian, Alaska Native	1/34, (2.9%)	–	
	Black/African American	1/34, (2.9%)	1/48, (2.6%)	
	Other	–	2, (5.3%)	
**Ethnicity (Hispanic)**		1/34, (4.2%)	8/48, (16.7%)	0.073
		**mean (sd)**	**mean (sd)**	***p*-value**
**Age**		41.7 (10.9)	38.1 (10.7)	0.12
**Headache Frequency**		20.4 (6.2)	16.1 (8.6)	**0**.**015**
**Years w/headache**		24.9 (14.4)	10.6 (8)	**<0**.**001**
**ASC**		5 (4.1)	5.6 (5.2)	0.895
**BDI**		9 (6)	17.1 (8.9)	**<0**.**001**
**COMPASS31**		27.3 (12.9)	36.6 (14.4)	**0**.**006**
**Hyperacusis**		14.4 (8.5)	23.3 (10.7)	**<0**.**001**
**Insomnia**		18.3 (6.8)	23.5 (6.6)	**0**.**002**
**MIDAS**		48.4 (33.5)	67.9 (59.8)	0.305
**PCS**		19.9 (10.9)	24.4 (13.2)	0.155
**State Anxiety**		34.8 (8.9)	37.7 (13.4)	0.498
**Trait Anxiety**		40 (10.3)	45 (13.4)	0.062
**Photophobia**		0.49 (0.29)	0.61 (0.33)	0.080

ASC, allodynia Symptom checklist; BDI, beck depression index; COMPASS, composite autonomic symptom score; MIDAS, migraine disability assessment; PCS, pain catastrophizing scale; *n*, sample size.

Thirty subjects with migraine had 15 or more headache days per month (chronic migraine). Twenty-eight of the PPTH subjects had 15 or more headache days per month. Forty-six of the PPTH participants had a migraine-like phenotype, with the other two displaying a tension-type headache-like phenotype. On the day of the visit 26/34 (76.4%) migraine participants and 42/48 (87.5%) PPTH participants reported a current pain level greater than zero (*p* = 0.8226). Presence of headache at time of imaging was not recorded.

### Classification models

**Figure 1:**
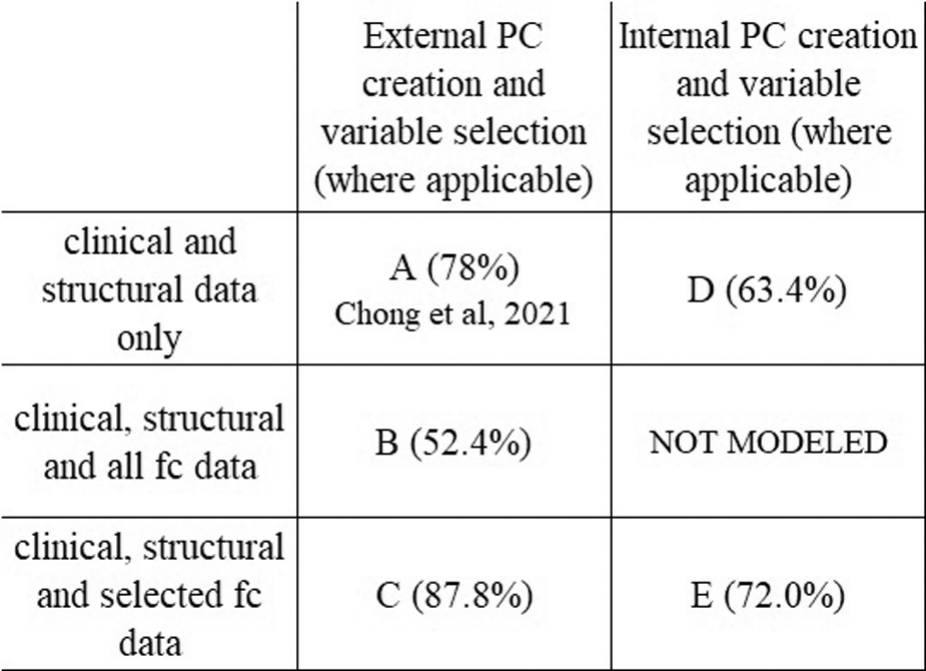
Model comparison. Figure shows the relationship between the classification models and each model's average accuracy. Each approach utilizes the same data set. Model A is the model from Chong et al., 2021 (78.0%). All models include the same patients, and same questionnaire data, structural and DTI data as variables. There was no variable selection on clinical or structural data in any model. The improvement in classifier accuracy by including fc data is seen in the increase in accuracy from A to C and from D to E. The performance metrics of models with internal pc creation and variable selection (**D,E**) are more conservative than the models with external pc creation and variable selection (**A,B,C**).

The number of potential variables by category are shown in Table 2. All classification models described here include all of the same questionnaire data, structural imaging data, and DTI data as variables. Each classifier model created 65 PCs from those variables which were used as variables in the model's ridge regression. The classification models differ in which fc data is included and whether the PC creation occurred inside or outside of the cross-validation loop. Showing the different models illustrates the benefit of including functional connectivity data; in the way the model was created in the previous publication (Figure 1A–C) and in a more conservative approach to model appraisal where the PC creation and variable selection happen inside the cross-validation loop (Figure 1D,E). In Figure 1 A is the model from the previous publication, B and C add fc data to A. Model D uses the same data as A but with PC creation inside the cross-validation loop. Model E adds fc data to D. The same subjects are used in all cases.

**Table 2:** Candidate Variables by Source.

Data Source	Imaging Sequence	Description	Candidate Variables
Clinical Data	n/a	Questionnaire Data	284
Cortical Volume	T1	Desikan Atlas regional Volume	134
Cortical Thickness	T1	Desikan Atlas regional thickness	70
Cortical Area	T1	Desikan Atlas regional area	70
Brain Curvature	T1	Desikan Atlas regional curvature	68
Fibertract	DTI	Axial, radial, and mean diffusivity, fractional anisotrophy, length and volume for 18 fibertracts	306
Static FC	Resting State fMRI	Region-to-region static fc	2346
Dynamic FC	Resting State fMRI	Region-to-region dynamic fc	2346
Total			5624

#### Models with PC creation outside the cross-validation loop

When no fc data is included, and PC creation is outside of the cross-validation loop, average model accuracy is 78.0%. If all fc data is included (no variable selection), then average model accuracy is 52.4%. When variable selection of fc data prior to PC creation is implemented the number of included static fc variables decreases to 144 and the number of included dynamic fc variables to 166. The resulting classifier has an average accuracy of 87.8%.

#### Models with pc creation inside the cross-validation loop

To address issues potentially arising from performing variable selection outside of the cross-validation loop, we performed an additional analysis where the variable selection and PC creation was moved inside the cross-validation loop. Doing this provides a more conservative appraisal of the generalizability of the model. In these models the average accuracy with fc data is 72.0% compared to 63.4% without the fc data.

Because it is more conservative, the classification model with fc variable selection and PC creation inside the cross-validation loop was used as the basis of all further analysis in this document. The confusion matrix for this model is shown in Table 3.

**Table 3:** Confusion Matrix for Model with Internal Variable Selection and PC Creation Including FC Data.

	Predicted Class	
Actual Class	Migraine	PPTH
Migraine	24	10
PPTH	13	35

Matrix shows the number of migraine subjects correctly (24) and incorrectly (10) classified and the number of PPTH subjects correctly (35) and incorrectly (13) classified by the model.

Balanced accuracy for this model is 71.8%. 72.9% of PPTH patients and 70.6% of migraine patients were correctly classified.

### Variable importance

Of the 5,624 unique candidate variables, 1,521 were selected for inclusion across one or more of the cross-validation loops in the internal variable selection model, including 310 static fc variables and 287 dynamic fc variables. Of those, 101 static fc and 103 dynamic fc variables were selected in every cross-validation loop.

Of the highest contributing 100 variables in the internal variable selection approach, 24 are fiber tract/DTI data, 22 are static fc and 18 are dynamic fc (Table 4).

**Table 4:** Number of Variables in the Top 100 Most Contributing; by Source.

Source	N
Clinical Data	9
Cortical Volume	8
Cortical Thickness	7
Cortical Area	0
Brain Curvature	12
Fibertract	24
Static fc	22
Dynamic fc	18

Variables in internal PC with selected fc data model.

**Figure 2:**
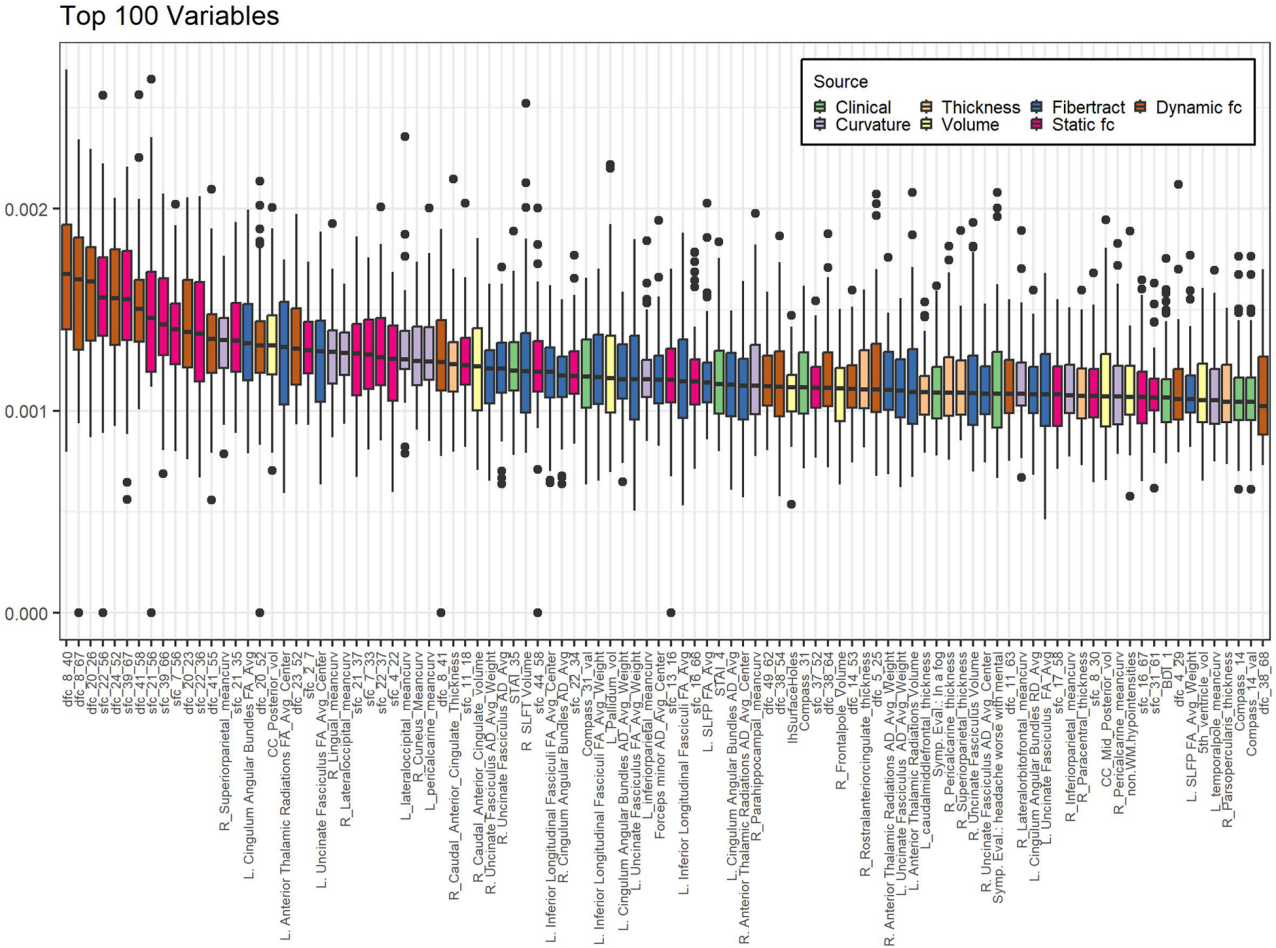
Variables contributing most to internal variable selection model. Internal PC with selected fc data model. Individual data points represent the contribution of a single variable to the model created in a single-cross validation loop. Data is grouped by variable to form a single boxplot for each variable on the plot. Bar indicates median, box ends at 1st and 3rd quartiles, whiskers show extent of non-outlier data. Dots are outliers. Boxes are sorted in order of decreasing ranking (mean contribution). Calculation of contribution is described in the methods section. Sfc: static functional connectivity; dfc: dynamic functional connectivity; R: right; L: left; mean_curv: mean curvature; vol: volume; RD: radial diffusivity; SLFP: superior longitudinal fasciculi - parietal; SLFT: superior longitudinal fasciculi – temporal; Symp. Eval.: Symptom Evaluation. See supplemental information for functional connectivity region number descriptions.

Figure 2 shows boxplots of contribution of the top 100 most contributing variables in the internal variable selection and PC creation model, ordered by ranking. While fiber tract data has more variables in the top 100 (24) than static fc (22) or dynamic fc (18) all the top ten most contributing variables are static or dynamic fc.

#### Static functional connectivity

**Figure 3:**
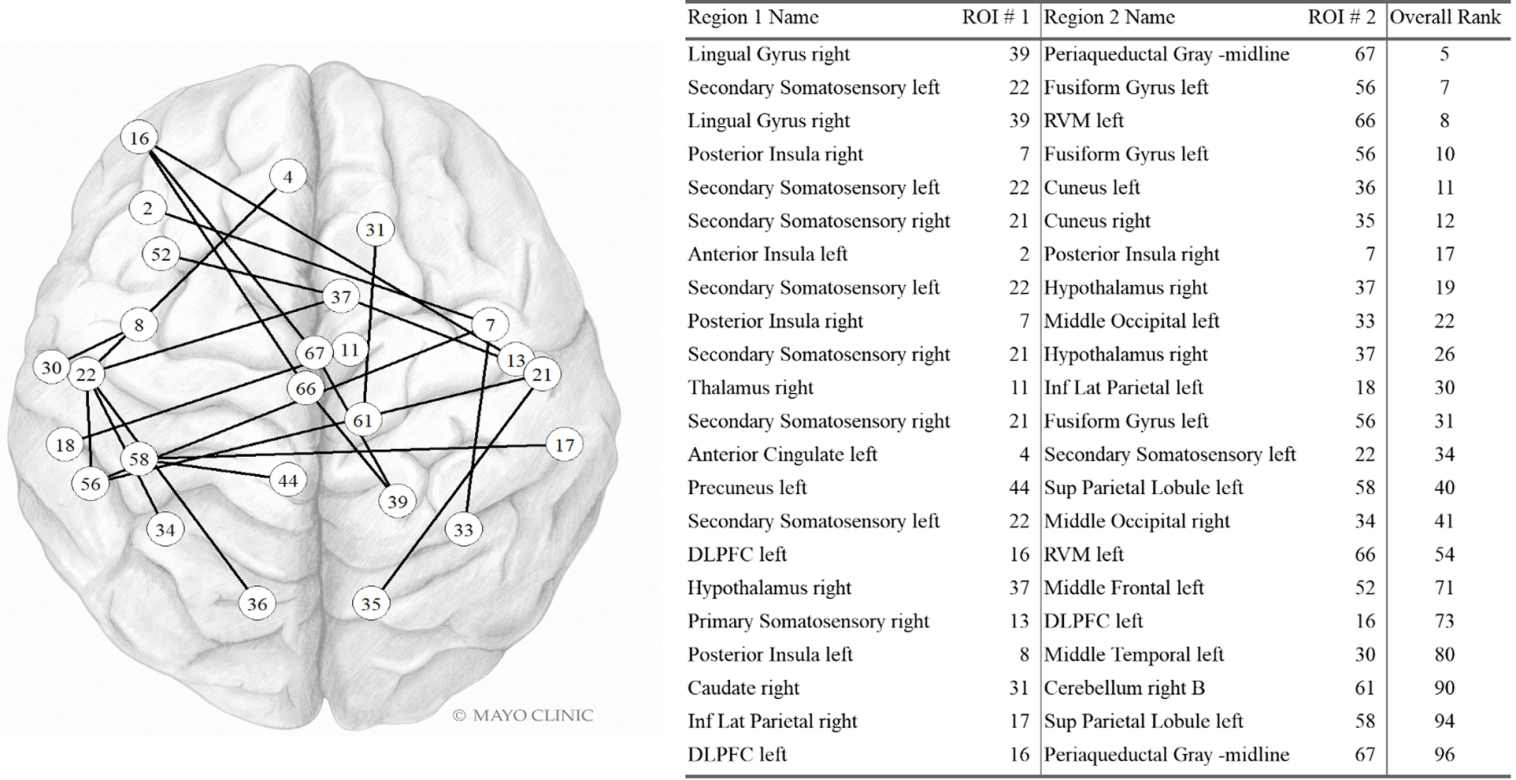
Static FC variables in the Top 100. Internal PC with selected fc data model. Network vertices labeled by ROI number as in Supplementary Table S1, Appendix. Vertex positions are approximate. DLPFC: Dorsolateral prefrontal cortex; Inf Lat Parietal: Inferior lateral. Used with permission of Mayo Foundation for Medical Education and Research. All rights reserved.

Sfc pairs found in the highest contributing 100 variables are shown in Figure 3. Five of the 22 most contributing static fc variables involve the left secondary somatosensory region and three involve the right somatosensory region. The right posterior insula, left DLPFC, right hypothalamus, and left fusiform gyrus are also part of three pairwise connections each. The right lingual gyrus, periaqueductal gray, left rostral ventromedial medulla, and left superior parietal lobule are each found twice while the remaining regions are found only once.

#### Dynamic functional connectivity

**Figure 4:**
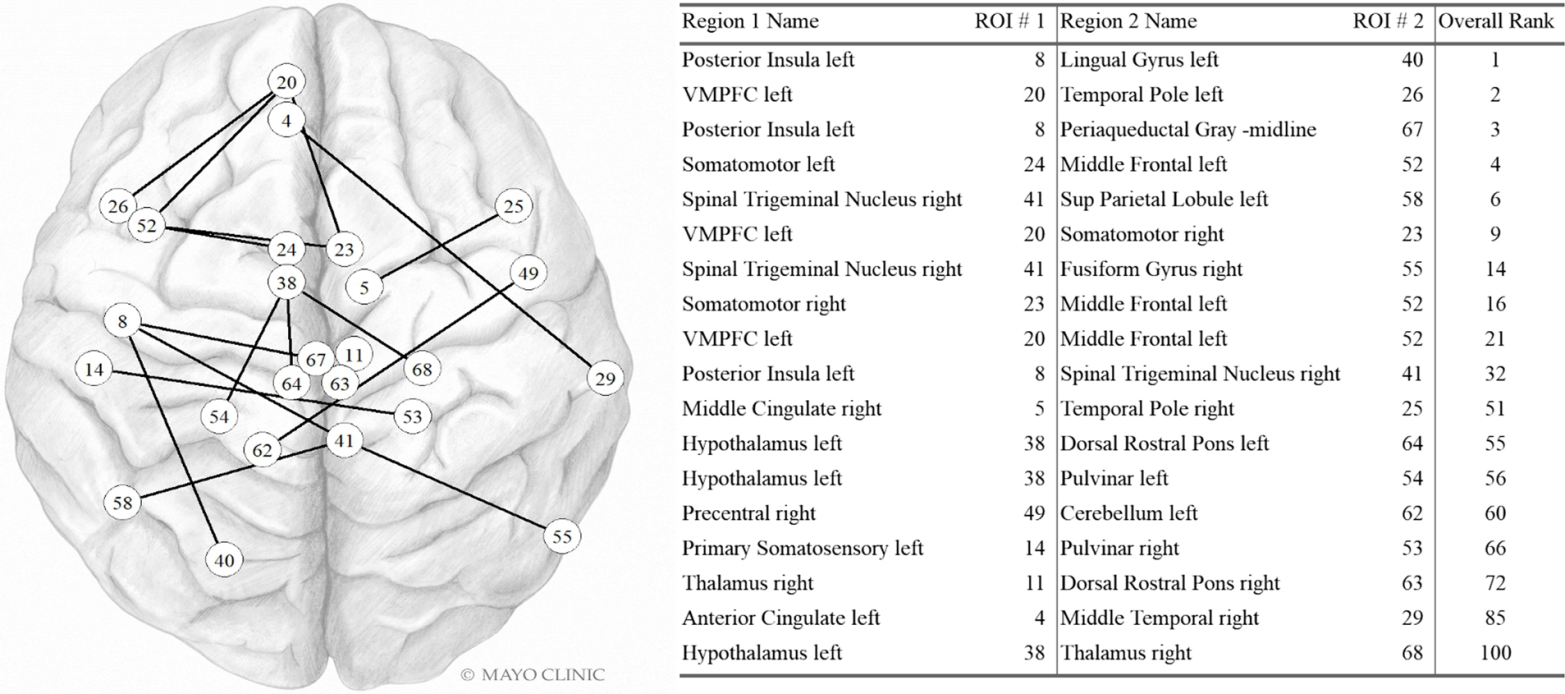
Dynamic FC variables in the Top 100. Internal PC with selected fc data model. Network vertices labeled by ROI number as in Supplementary Table S1, Appendix. Vertex positions are approximate. VMPFC: ventromedial prefrontal cortex; Sup Parietal: Superior Parietal. Used with permission of Mayo Foundation for Medical Education and Research. All rights reserved.

Dfc pairs found in the highest contributing 100 variables are shown in Figure 4. The left posterior insula, left VMPFC, right spinal trigeminal region, left hypothalamus, and left middle frontal were each part of three dynamic fc pairwise connections in the top 100 variables. The right somatomotor was involved in two dynamic fc pairwise connections in the top 100 variables. The other regions in this list were involved in a single pairwise dynamic fc connection in the top 100 variables.

#### Differences between correctly and incorrectly classified

**Figure 5:**
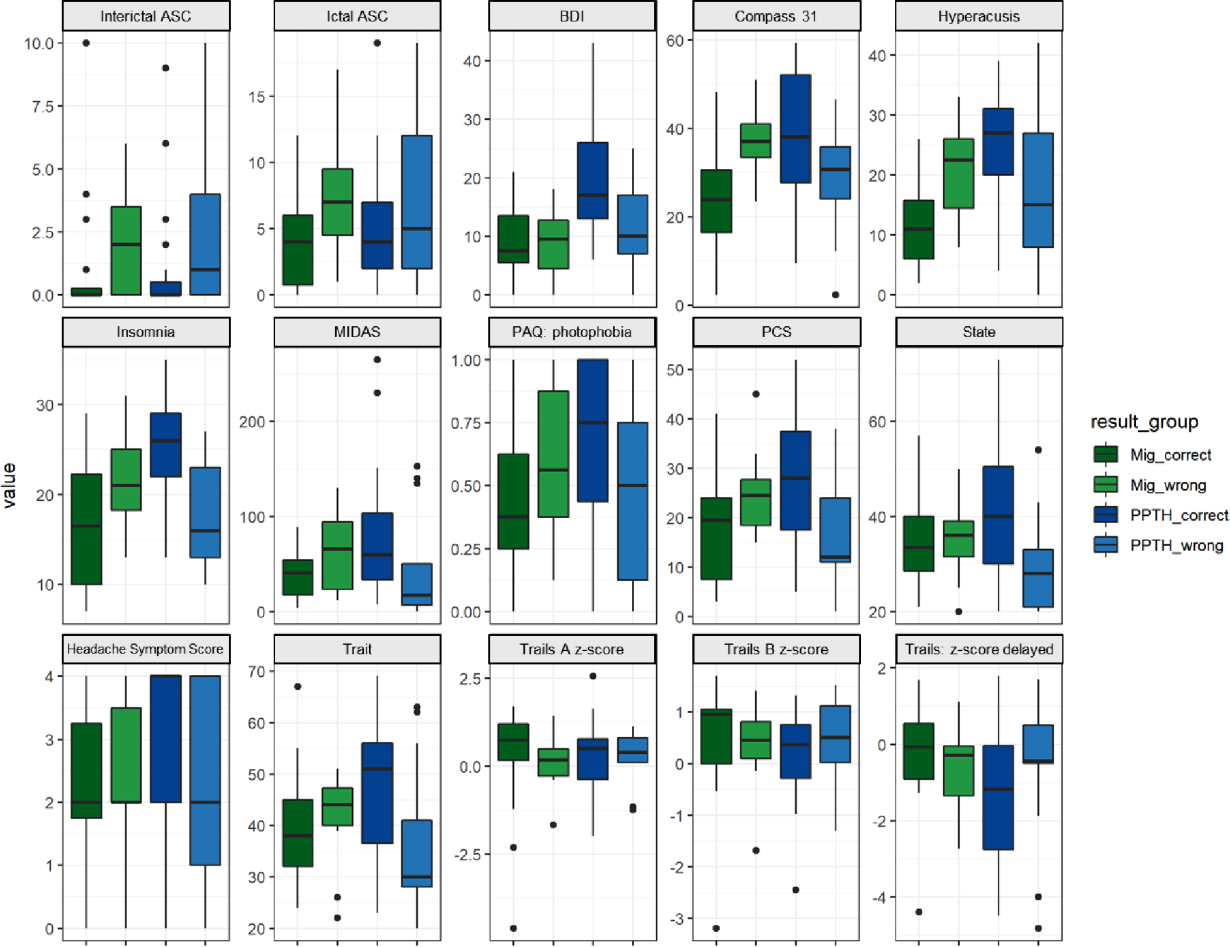
Instrument scores by correctly vs incorrectly classified migraine vs PPTH groups. Internal PC with selected fc data model. ASC = Allodynia Symptom Checklist; BDI = Beck Depression Inventory; COMPASS = Composite Autonomic Symptom Score; MIDAS = Migraine Disability Assessment; PAQ = Photosensitivity Assessment Questionnaire; State = state anxiety; Trait = trait anxiety. Dots are outliers.

In general, the misclassified PPTH group was less severely affected than the correctly classified PPTH group. The incorrectly classified PPTH group had lower median BDI, COMPASS 31, hyperacusis, insomnia, MIDAS, photophobia, PCS, state anxiety, trait anxiety and headache symptom score than the correctly classified PPTH group. (Figure 5). Median allodynia scores (ASC) were higher for the misclassified PPTH group than the correctly classified PPTH group. The opposite pattern is repeated for the misclassified migraine group compared to the correctly classified migraine group but to a lesser degree. The exception is that the incorrectly classified migraine group had higher median allodynia than the correctly classified migraine group. It is not surprising to find the correctly classified cases at the extremes with the incorrectly classified groups between them; the cases that look most like the other group will be the hardest to distinguish. However, large differences are interesting because while the variables shown here were included in the model, they weren't found among the most influential variables.

Sex was included as a candidate variable in each model but was not found to have high importance in the final model (see Table 5). Males were more likely than females to be correctly categorized, especially within the PPTH group (Chi-sq, *p* = 0.008).

**Table 5:** Sex Breakdown Correctly vs Incorrectly Classified.

	Migraine			PPTH		
	Sex					
	Female	Male	% Male	Female	Male	% Male
Correct	14	10	41.7%	8	27	77.1%
Incorrect	8	2	20.0%	9	4	30.8%
% Correct	63.6%	83.3%		47.1%	87.1%	

Internal PC with selected fc data model.

There were no apparent differences between the correctly and incorrectly classified subjects in terms of pain intensity, headache duration, frequency, or years with headache. This suggests their inclusion in the model would be unlikely to improve the performance.

Correctly and incorrectly classified subjects were equally likely to have taken medication in the last 48 h (Table 6) (*p* = 0.23, Chi-squared), suggesting having done so does not substantially impact imaging findings included in this analysis. Those with migraine were more likely to have reported a family history of migraine but it did not relate to classification success. A greater percentage of PPTH subjects who had aura were correctly classified compared to those who did not have aura, although aura was not included as a candidate variable. The two PPTH subjects with a tension-type headache phenotype were both correctly classified.

**Table 6:** Group Differences for Discrete Characteristics Not Included in the Classification Models.

	Migraine	PPTH
	**Handedness**					
	right	left	% left	right	left	% left
Correct	22	2	8.3%	33	2	5.7%
Incorrect	8	2	20.0%	12	1	7.7%
% Correct	73.3%	50.0%		73.3%	66.7%	
	**Taking Preventive Medications**					
	No	Yes	% Yes	No	Yes	% Yes
Correct	12	12	50.0%	16	19	54.3%
Incorrect	6	4	40.0%	9	4	30.8%
% Correct	66.7%	75.0%		64.0%	82.6%	
	**Meds in last 48 hours**					
	No	Yes	% Yes	No	Yes	% Yes
Correct	11	12	52.2%	16	19	54.3%
Incorrect	5	5	50.0%	6	6	50.0%
% Correct	68.8%	70.6%		72.7%	76.0%	
	**Migraine Family History**					
	No	Yes	% Yes	No	Yes	% Yes
Correct	7	17	70.8%	29	5	14.7%
Incorrect	2	8	80.0%	11	2	15.4%
% Correct	77.8%	68.0%		72.5%	71.4%	
	**Aura**					
	No	Yes	% Yes	No	Yes	% Yes
Correct	12	12	50.0%	16	19	54.3%
Incorrect	4	6	60.0%	9	4	30.8%
% Correct	75.0%	66.7%		64.0%	82.6%	
	**Headache Phenotype**					
				Migraine	Probable migraine	TTH
Correct	Migraine			28	7	0
Incorrect				9	2	2
% Correct				75.7%	77.8%	0.0%

Internal PC with selected fc data model; correctly vs incorrectly classified, Migraine vs PPTH. Meds in last 48 hours = individuals who had taken a pain medication or acute medication for treatment of headache within the prior 48 hours.

## Discussion

Adding static and dynamic fc data to a machine learning classifier based on clinical and structural data can improve classifier ability to distinguish individuals with migraine from those with PPTH. The average accuracy of our previously published model was 78.0%. However, this study showed that the addition of pre-selected functional connectivity data increased model accuracy to 87.8%. For the models with variable selection and PC creation inside the cross-validation loop the addition of fc data raised the average accuracy from 63.4% to 72.0%. Within each of these pairings (78% vs. 87.8% and 63.4% vs. 72.0%) the models being compared are directly comparable; the inclusion of fc data is the only difference, so we can confidently assert that inclusion of fc data improves the model performance. The model with variable selection and PC creation outside of the cross-validation represents a more optimistic view while the model with variable selection and PC creation on the inside represents a more conservative view.

When it is difficult to find/create a data set with the necessary variables that is large enough to allow for a separate held-out test set, cross-validation is frequently used. Cross-validation allows a data set to be used for both model training and evaluation (non-simultaneously) as well as variable selection, but the use of the data in all of these roles concurrently can lead to bias in the model and overestimation of model performance. The substantial difference in performance (∼16%) between the internal and external models, which were built with the same general method on the same data set, illustrates the importance of understanding how classification model accuracy is reported in the literature. By presenting both the internal and external model here we can have a better understanding of the potential range of performance we would be likely to see if the modeling was used to classify new groups of people with PPTH and migraine.

Given the improvement to model accuracy with inclusion of fc data it is not surprising that many of the most important variables are tied to fc. Regions that were important contributors to the model included those located within somatosensory cortex, posterior insula, prefrontal cortex, hypothalamus, periaqueductal gray, rostral ventral medulla, fusiform gyrus, and lingual gyrus. These are regions that participate in different aspects of the migraine and PPTH experience including sensory-discriminative and cognitive processing of pain (31), pain modulation (32, 33), migraine attack generation (34), and multisensory integration (35). These regions have previously been demonstrated to have atypical function amongst individuals with PPTH and/or migraine (36–40). Precise explanations for why the functional connections between specific brain regions were important for differentiating migraine and PTH are speculative. The hypothalamus is known to be involved in chronic pain, including migraine and PTH. Altered activity and functional connectivity of the hypothalamus has been identified early in the migraine attack, suggesting the hypothalamus plays a role in migraine attack generation (41–44). It has also been implicated in the premonitory and headache phases of migraine (32, 45). The role of the hypothalamus in PTH is less certain, although there is emerging literature suggesting the hypothalamus is involved in PTH. For example, a sfc study of PTH demonstrated that altered connectivity between the hypothalamus and frontal lobe correlated with headache frequency and intensity (46). In the analysis reported herein, the hypothalamus was involved with several static and dynamic functional connections that provided important contributions to the classification task. Similarly, the secondary somatosensory region was included in multiple highly contributing sfc connections. This might be expected based on its role in the processing and integration of painful and non-painful somatosensory stimuli, prior studies demonstrating alterations in responses to tactile stimulation in migraine and PTH, prior evidence for atypical pain-induced secondary somatosensory cortex activation in people with migraine and ictal allodynia, and atypical sfc of secondary somatosensory cortex in those with PTH (47–50). Since functional connectivity of the hypothalamus and secondary somatosensory cortex contributed to differentiating migraine and PTH, the contributions of these regions to the pathophysiology of these two distinct headache types might differ; further studies are needed to better explain these differences.

The variable ranked as most important for classification was the dfc between the posterior insula and the left lingual gyrus. These brain regions are known to be involved in the processing of somatic sensations and visual processing respectively. Several prior studies have demonstrated structural and functional alterations in insula and the visual network in migraine (51–53). Their inclusion together here could be indicative of differences in brain function related to visual hypersensitivities and integration of visual and somatosensory stimuli (e.g., photo-allodynia, worsening of headache when exposed to visual stimuli), a known phenomenon in both migraine and PTH. Of note, photophobia questionnaire scores did not differ by group and were not included as top ranked variables themselves. It is possible that the inclusion of this dynamic functional connectivity information, in combination with the other information captured across the included principal components, is telling us something more nuanced about migraine vs. PTH differences in photosensitivity or other forms of visual hypersensitivity than is captured by the photophobia questionnaire.

The top ranked sfc input was between the right lingual gyrus and the periaqueductal gray. The periaqueductal gray is an important region for pain modulation. Numerous prior studies have identified atypical structure and function of the periaqueductal gray in chronic pain and migraine (54–57). A PTH study demonstrated altered sfc of the periaqueductal gray in patients who were imaged within one week of their brain injuries, and the periaqueductal gray connectivity helped to predict PTH persistence at three months (58). Activation of the lingual gyrus has also been shown to vary between individuals with migraine with simple visual aura and those with complex aura (59). Our study populations were both equally likely to report the presence of aura, but the inclusion of the lingual gyrus in multiple highly ranked fc variables may reflect a more subtle difference in the type of aura experienced in each group.

Identification of regions that most contribute to differentiating migraine and PTH assist with choosing which brain regions to further interrogate in future studies interested in pathophysiological differences between migraine and PTH. These future studies might also be able to provide greater insights into clinical characteristics of migraine and PTH that associate with aberrant functional connectivity of these regions.

The internal PC model with fc data had nearly equal accuracy between the two classes; 70.6% of migraine and 72.9% of PPTH subjects were correctly classified. Positive predictive value for identifying PPTH subjects was 77.8%. This contrasts with the previous publication where 97.7% of the migraine group but only 64.6% of the PPTH group were correctly classified. This result further supports the importance of including fc data for PPTH classification.

### Limitations

Our fMRI set up did not include collection of pulse, breathing or blood pressure measurements, nor did we account for spontaneous blood pressure changes that may occur at the upper limit of the frequency band (60), which are study limitations.

Machine learning models are sensitive to the choice of hyperparameters; variables which control the model's learning process. In the models reported here, the number of PCs, the ridge regression regularization parameter, and the parameters controlling the inclusion and exclusion of candidate variables are hyperparameters which must be set. The set of regularization parameter candidate values, the use of 65 PCs, and the use of *p *<=* *0.05 in variable selection may not be absolutely optimal. Future studies should include individuals with PPTH with history of migraine pre-mTBI. Sex was accounted for by its inclusion as a model variable however future studies would benefit by utilizing cohorts that are more closely matched in sex and years lived with headache.

Similarly, the choice of sliding window length when calculating the dfc may affect the outcome. While we have made every effort to make the models directly comparable, we have not shown the statistical significance of the improvement in model accuracy due to the addition of the fc data.

We did not account for race and ethnicity in this analysis. The homogeneity of race and ethnicity overall as well as the uniformity of race and ethnicity across groups (PPTH and healthy controls) makes it unlikely that these variables influenced the outcome of this analysis. The small numbers of non-White and Hispanic study participants makes sub-analysis infeasible.

### Future work

In the future, a relatively objective method for differentiating PPTH from migraine might increase confidence in the diagnosis when doing so is difficult on clinical grounds alone. A classifier would be particularly useful when details about the TBI and timing of headache symptoms are less clear. Currently, accurate differentiation is particularly important when enrolling subjects into clinical trials; it will also be important when recommending treatment as soon as PTH-specific treatments are available.

## Conclusions

A classification model based on clinical questionnaire data, structural imaging features and fc features can differentiate those with PPTH from those with migraine with 87.8% accuracy. The inclusion of fc data improved the accuracy of the model from the previously published 78%.

A more conservative method with variable selection and PC creation inside the cross-validation loop showed 72% accuracy when fc data were included, compared to 63.4% when fc data were not included. Fc and fibertract data were important contributors to the classifier. Classification accuracy was approximately equal for classifying PPTH and migraine.

## Data Availability

The original contributions presented in the study are included in the article/**Supplementary Materials**, further inquiries can be directed to the corresponding author/s.
