# Supplementary material for: The value of brain MRI functional connectivity data in a machine learning classifier for distinguishing migraine from persistent post-traumatic headache
Source: Front Pain Res (Lausanne). 2023 Jan 9;3:1012831. doi: 10.3389/fpain.2022.1012831 (PMC9869115; doi:10.3389/fpain.2022.1012831)
Supplement: Supplementary file 1 [file Datasheet1.docx]

APPENDIX

Appendix Table 1: Functional Connectivity ROI Coordinates.

| **ROI** | **Region Name** | **x** | **y** | **z** |
| --- | --- | --- | --- | --- |
| 1 | Anterior Insula right | 38 | 19 | -3 |
| 2 | Anterior Insula left | -38 | 19 | -3 |
| 3 | Anterior Cingulate right | 6 | 28 | 24 |
| 4 | Anterior Cingulate left | -6 | 28 | 24 |
| 5 | Middle Cingulate right | 10 | -7 | 46 |
| 6 | Middle Cingulate left | -10 | -7 | 46 |
| 7 | Posterior Insula right | 40 | -14 | 1 |
| 8 | Posterior Insula left | -40 | -14 | 1 |
| 9 | Posterior Cingulate right | 8 | -48 | 39 |
| 10 | Posterior Cingulate left | -8 | -48 | 39 |
| 11 | Thalamus right | 8 | -21 | 7 |
| 12 | Thalamus left | -8 | -21 | 7 |
| 13 | Primary Somatosensory right | 46 | -24 | 47 |
| 14 | Primary Somatosensory left | -46 | -24 | 47 |
| 15 | DLPFC right | 40 | 39 | 24 |
| 16 | DLPFC left | -40 | 39 | 24 |
| 17 | Inf Lat Parietal right | 57 | -48 | 30 |
| 18 | Inf Lat Parietal left | -57 | -48 | 30 |
| 19 | VMPFC right | 6 | 36 | -14 |
| 20 | VMPFC left | -6 | 36 | -14 |
| 21 | Secondary Somatosensory right | 52 | -28 | 21 |
| 22 | Secondary Somatosensory left | -52 | -28 | 21 |
| 23 | Somatomotor right | 6 | 1 | 68 |
| 24 | Somatomotor left | -6 | 1 | 68 |
| 25 | Temporal Pole right | 41 | 10 | -32 |
| 26 | Temporal Pole left | -41 | 10 | -32 |
| 27 | Amygdala right | 22 | -1 | -22 |
| 28 | Amygdala left | -22 | -1 | -22 |
| 29 | Middle Temporal right | 60 | -26 | -5 |
| 30 | Middle Temporal left | -60 | -26 | -5 |
| 31 | Caudate right | 14 | 13 | 11 |
| 32 | Caudate left | -14 | 13 | 11 |
| 33 | Middle Occipital left | 34 | -72 | 6 |
| 34 | Middle Occipital right | -34 | -72 | 6 |
| 35 | Cuneus right | 13 | -93 | 9 |
| 36 | Cuneus left | -13 | -93 | 9 |
| 37 | Hypothalamus right | 6 | -6 | -12 |
| 38 | Hypothalamus left | -6 | -6 | -12 |
| 39 | Lingual Gyrus right | 19 | -64 | -11 |
| 40 | Lingual Gyrus left | -19 | -64 | -11 |
| 41 | Spinal Trigeminal Region right | 6 | -39 | -45 |
| 42 | Spinal Trigeminal Region left | -6 | -39 | -45 |
| 43 | Precuneus right | 6 | -58 | 46 |
| 44 | Precuneus left | -6 | -58 | 46 |
| 45 | Parieto-Occipital right | 51 | -64 | 18 |
| 46 | Parieto-Occipital left | -51 | -64 | 18 |
| 47 | Supramarginal Gyrus right | 44 | -42 | 24 |
| 48 | Supramarginal Gyrus left | -44 | -42 | 24 |
| 49 | Precentral right | 44 | -4 | 40 |
| 50 | Precentral left | -44 | -4 | 40 |
| 51 | Middle Frontal right | 35 | 6 | 52 |
| 52 | Middle Frontal left | -35 | 6 | 52 |
| 53 | Pulvinar right | 20 | -34 | 3 |
| 54 | Pulvinar left | -20 | -34 | 3 |
| 55 | Fusiform Gyrus right | 51 | -59 | -9 |
| 56 | Fusiform Gyrus left | -51 | -59 | -9 |
| 57 | Sup Parietal Lobule right | 40 | -52 | 49 |
| 58 | Sup Parietal Lobule left | -40 | -52 | 49 |
| 59 | Cerebellum right A | 20 | -46 | -50 |
| 60 | Cerebellum left A | -20 | -46 | -50 |
| 61 | Cerebellum right B | 11 | -41 | -19 |
| 62 | Cerebellum left B | -11 | -41 | -19 |
| 63 | Dorsal Rostral Pons right | 5 | -27 | -29 |
| 64 | Dorsal Rostral Pons left | -5 | -27 | -29 |
| 65 | RVM right | 2 | -32 | -49 |
| 66 | RVM left | -2 | -32 | -49 |
| 67 | Periaqueductal Gray midline | -1 | -26 | -11 |
| 68 | Thalamus right B | 22 | -24 | 0 |
| 69 | Thalamus left B | -22 | -28 | 6 |

DLPFC = dorsolateral prefrontal cortex; Inf = inferior; Lat = lateral; VMPFC = ventromedial prefrontal cortex; sup = superior; RVM = rostral ventral medulla. X, y, z coordinates based on MNI template.

## Appendix: Imaging Details

MRI-based Imaging:

Trained Mayo Clinic Radiology technologists, assisted by research study personnel, acquired imaging sequences. Subjects were scanned over a 26-month period (2016-2018). Imaging sequences were collected using a 20-channel head/neck coil.

MRI sequence collection included the following: **3D T1-weighted sagittal MP-RAGE** (TE=3.03ms, TR=2400ms, flip angle=8°), 128 slices, slice thickness=1.25mm (no gap), voxel dimensions=1mmx1mm, field of view (FOV)= 256mm^2^ matrix size=256x256. Axial T2-weighted imaging: (TE=84ms, TR=6800ms, flip angle=150°), 38 slices, voxel dimensions=1x1x4 mm^3^, slice thickness=4mm, FOV=256mm^2^, matrix size=256x256. **Diffusion Tensor Imaging** (DTI): (TE=73ms, TR=5200ms, flip angle=90°), 38 slices, slice thickness= 4mm, voxel dimensions=1.7x1.7x4 mm^3^, FOV=220mm^2^, matrix size=128x128. 30 diffusion weighted (b=1000 s/mm^2^) non-linear directions and one image without diffusion weighting (diffusion-unweighted volume; b=0s/mm^2^). T2-weighted imaging and diffusion tensor imaging were acquired with two averages. **Blood oxygenation level dependent (BOLD) resting-state sequence**: (TE=27ms, TR =2500 ms, flip angle=90°), voxel dimensions= 4 × 4 × 4 mm^3^, FOV=256mm^2^, 118 volumes of 38 slices.

MRI data preprocessing for T1-weighted data: All structural imaging data were preprocessed using FreeSurfer, analysis suite (version 6.0) and the integrated automated global tractography toolbox TRACULA (TRActs Constrained by Underlying Anatomy)[1]. Preprocessing of anatomical T1-weighted imaging was conducted using the automated ‘recon-all’ surface-based brain segmentation and parcellation stream and the Desikan-Killiany Atlas which segments each subjects T1-weighted brain data into 34 regions in each hemisphere for the estimation of cortical thickness, volume, area, and curvature [2]. Data processing steps of this technique include: removal of non-brain tissue (skull stripping) [3], Talairach transformation [4], segmentation and parcellation of gray and white matter, intensity normalization and brain boundary tessellation [5], and surface deformation [6].

MRI data preprocessing for DTI data: The FreeSurfer TRACULA package reconstructs 18 major white matter pathways and associated diffusion metrics (fractional anisotropy, mean diffusivity, axial diffusivity, and radial diffusivity). The fibertracts include: the forceps major and forceps minor tracts, bilateral corticospinal tract, bilateral uncinate fasciculus, bilateral cingulum cingulate gyri, bilateral superior longitudinal fasciculi-parietal*,* bilateral anterior thalamic radiations, bilateral cingulum angular bundles, bilateral superior longitudinal fasciculi- *temporal*, and bilateral inferior longitudinal fasciculi.

All white matter tracts were reconstructed automatically using each individual’s T1 anatomical priors. TRACULA uses probabilistic tractography from FSL for the standard preprocessing of diffusion data. Steps include image correction to correct for B0 inhomogeneities, eddy current and head motion correction, brain extraction using ‘bet’ and ‘bedpostx’ to fit the ball-and-stick model for calculating voxel-based diffusion parameters. Bvecs are rotated for eddy current correction. Within-subject registration is performed by affine registration of each subject’s low-b diffusion to the individual’s T1-weighted imaging and then co-registering to a template for between group comparisons.

Diffusion gradient directions were manually checked for correctness in ‘fslview’ before including subject data in the final analysis. In combination with a pre-labeled training set, the structural brain anatomy of each person was used to estimate the distribution of fibertracts using a Markov Chain Monte Carlo algorithm [1]. All image preprocessing was conducted on a single Mac workstation (3.5 Ghz 6-core Xeon E5) installed with OS X Sierra (10.12.6) to prevent preprocessing irregularities stemming from using multiple workstations [7]. A neuroimaging scientist (CC) manually inspected the accuracy of the brain segmentation output before including subject data for group analyses to ensure the accuracy of the automated brain reconstruction process and prevent the inclusion of erroneous datasets. All imaging was assessed for excessive motion, and subjects which moved >2mm in either direction during scanning were excluded from the final analysis. Additionally, between-groups differences (migraine vs PPTH) for movement in the scanner were assessed using two-sided t-tests, which showed no significant group differences in movement parameters (p=0.3).

MRI data preprocessing for Resting State Functional T1-weighted data:

All functional imaging was preprocessed using SPM 8 (Wellcome Department of Cognitive Neurology, Institute of Neurology, London, UK) and the SPM toolbox DPARSF [8], interfaced with MATLAB version11.0 (MathWorks, Natick, MA, USA).

Resting-state data were processed according to standard SPM methodology [9], which included the following: slice time correction, motion correction, and realignment to the first volume; skull and non-brain tissue were removed, and data were spatially smoothed to 6 mm, full width at half maximum (FWHM). Each participant’s resting-state images were aligned to their own T1-weighted scan and then transformed to the standardized Montreal Neurological Institute (MNI) 305 template to enable signal averaging across all participants. Data were bandpass filtered to between 0.01 to 0.1 Hz to capture low-frequency components [10]. Signals of no interest (including white matter signal, cerebrospinal fluid signal, and global mean signal) were regressed from the data. Variance due to head motion was regressed using a framewise displacement model [11]. Using the DPARSF head motion output file all scans were checked for motion, allowing the exclusion of data from participants that exceeded the predetermined motion limit (>2mm movement in scanner) from the final analysis.

A region of interest (ROI) approach was used to assess functional connectivity. Sixty-nine ROIs (34 bilateral regions and one midline area) were identified a priori (see Table 1, appendix) from prior literature showing that the brain regions a) have atypical fc or functional activation in prior pain-related studies (including migraine) or b) are typically implicated in pain processing [12–21]. Region names and x, y, and z coordinates are shown in Table 1, appendix. Coordinates were determined using the MNI atlas.

Static fc: Eight-millimeter spheres were drawn around ROIs 1-58 and 4mm spheres were drawn around ROIs 59-69. Time courses over each seed region were extracted, and Pearson correlation matrices were computed.

Dynamic fc: A sliding window correlation analysis was performed for each ROI pair, and the standard deviation of the resulting time course values were calculated. Window length was 60 seconds.

1. Yendiki A, Panneck P, Srinivasan P, et al (2011) Automated probabilistic reconstruction of white-matter pathways in health and disease using an atlas of the underlying anatomy. Front Neuroinform 5:1–12. https://doi.org/10.3389/fninf.2011.00023

2. Desikan RS, Ségonne F, Fischl B, et al (2006) An automated labeling system for subdividing the human cerebral cortex on MRI scans into gyral based regions of interest. Neuroimage 31:968–980. https://doi.org/10.1016/j.neuroimage.2006.01.021

3. Ségonne F, Dale AM, Busa E, et al (2004) A hybrid approach to the skull stripping problem in MRI. Neuroimage 22:1060–1075. https://doi.org/10.1016/j.neuroimage.2004.03.032

4. Fischl B, Salat DH, Busa E, et al (2002) Whole brain segmentation: Automated labeling of neuroanatomical structures in the human brain. Neuron 33:341–355. https://doi.org/10.1016/S0896-6273(02)00569-X

5. Fischl B, Liu A, Dale AM (2001) Automated manifold surgery: Constructing geometrically accurate and topologically correct models of the human cerebral cortex. IEEE Trans Med Imaging 20:70–80. https://doi.org/10.1109/42.906426

6. Dale AM, Fischl B, Sereno MI (1999) Cortical Surface-Based Analysis. Neuroimage 9:179–194. https://doi.org/10.1006/nimg.1998.0395

7. Gronenschild EHBM, Habets P, Jacobs HIL, et al (2012) The effects of FreeSurfer version, workstation type, and Macintosh operating system version on anatomical volume and cortical thickness measurements. PLoS One 7:. https://doi.org/10.1371/journal.pone.0038234

8. Chao-Gan Y, Yu-Feng Z (2010) DPARSF: A MATLAB toolbox for “pipeline” data analysis of resting-state fMRI. Front Syst Neurosci 4:1–7. https://doi.org/10.3389/fnsys.2010.00013

9. Ashburner J, Friston KJ (1999) Nonlinear Spatial Normalization Using Basis Functions. Hum Brain Mapp 7:254–266

10. Cordes D, Haughton VM, Konstantinos A, et al (2001) Frequencies Contributing to Functional Connectivity in the Cerebral Cortex in “Resting-state” Data. Am J Neuroradiol 22:1326–1333. https://doi.org/10.1007/s10854-014-1833-2

11. Power JD, Mitra A, Laumann TO, et al (2014) Methods to detect, characterize, and remove motion artifact in resting state fMRI. Neuroimage 84:320–341. https://doi.org/10.1016/j.neuroimage.2013.08.048

12. Amin FM, Hougaard A, Magon S, et al (2018) Altered thalamic connectivity during spontaneous attacks of migraine without aura: A resting-state fMRI study. Cephalalgia 38:1237–1244. https://doi.org/10.1177/0333102417729113

13. Chong CD, Gaw N, Fu Y, et al (2017) Migraine classification using magnetic resonance imaging resting-state functional connectivity data. Cephalalgia 37:828–844. https://doi.org/10.1177/0333102416652091

14. Mickleborough MJS, Ekstrand C, Gould L, et al (2016) Attentional Network Differences Between Migraineurs and Non-migraine Controls: fMRI Evidence. Brain Topogr 29:419–428

15. Tedeschi G, Russo A, Conte F, et al (2016) Increased interictal visual network connectivity in patients with migraine with aura. Cephalalgia 36:139–147. https://doi.org/10.1177/0333102415584360

16. Peyron R, Laurent B, Garcia-Larrea L (2000) Functional imaging of brain responses to pain. Neurophysiol Clin 30:263–288

17. Stankewitz A, Aderjan D, Eippert F, May A (2011) Trigeminal nociceptive transmission in migraineurs predicts migraine attacks. J Neurosci 31:1937–1943. https://doi.org/10.1523/JNEUROSCI.4496-10.2011

18. Moulton EA, Becerra L, Maleki N, et al (2011) Painful heat reveals hyperexcitability of the temporal pole in interictal and ictal migraine states. Cereb Cortex 21:435–448. https://doi.org/10.1093/cercor/bhq109

19. Duerden EG, Albanese MC (2013) Localization of pain-related brain activation: A meta-analysis of neuroimaging data. Hum Brain Mapp 34:109–149. https://doi.org/10.1002/hbm.21416

20. Wager TD, Atlas LY, Lindquist MA, et al (2013) An fMRI-Based Neurologic Signature of Physical Pain. N Engl J Med 368:1388–1397. https://doi.org/10.1056/nejmoa1204471

21. Schulte LH, Menz MM, Haaker J, May A (2020) The migraineur’s brain networks: Continuous resting state fMRI over 30 days. Cephalalgia 40:1614–1621. https://doi.org/10.1177/0333102420951465
